# Shortening of central venous catheter time in men with hematological and oncological diseases for prevention of central venous catheter-related bloodstream infections: a target-trial-emulation study

**DOI:** 10.1007/s15010-026-02888-7

**Published:** 2026-07-10

**Authors:** Oliver Kriege, Boris Böll, Nicole Brüder, Nicola Giesen, Julia Lanznaster, Susanne Mertins, Antrea Minti, Jan-Hendrik Naendrup, Martin Schmidt-Hieber, Ruth Seggewiss-Bernhardt, Philipp Weber, Kai Wille, Jens Panse, Marcus Hentrich, Enrico Schalk, SECRECY Investigators, Sabine Einhell, Eva Fiegle, Pierre Kremer, Timo Schmitt, Sebastian Schulz, Christoph Schüssler, Daniela Tölle, Benedikt W. Pelzer, Judit Grans-Siebel

**Affiliations:** 1 Department of Hematology and Medical Oncology, University Medical Center of the Johannes Gutenberg University Mainz, Langenbeckstr. 1, 55131 Mainz, Germany; 2 Department of Internal Medicine I, University Hospital of Cologne, Cologne, Germany; 3 Department of Hematology, Hemostaseology, Oncology and Stem Cell Transplantation, Hannover Medical School, Hannover, Germany; 4 Department of Hematology, Oncology and Palliative Care, Robert Bosch Hospital, Stuttgart, Germany; 5 Department of Internal Medicine II, Passau Hospital, Passau, Germany; 6 Department of Internal Medicine II, Schwarzwald – Baar – Klinikum, Villingen-Schwenningen, Germany; 7 Department of Oncology, Hematology, Hemostaseology and Stem Cell Transplantation, University Hospital RWTH Aachen, Aachen, Germany; 8 Center for Integrated Oncology (CIO), Aachen, Bonn, Cologne, Düsseldorf (ABCD), Germany; 9 Clinic of Hematology, Oncology, Pneumology, Nephrology and Diabetology, Medical University Lausitz – Carl Thiem (MUL-CT), Cottbus, Germany; 10 Department of Hematology and Oncology, Sozialstiftung Bamberg, Bamberg, Germany; 11 Comprehensive Cancer Center Mainfranken (CCC MF), University Hospital of Würzburg, Würzburg, Germany; 12 Department of Internal Medicine I, Klinikum Mutterhaus der Borromaerinnen, Trier, Germany; 13 Oncology, Hemostaseology and Palliative Care, University Clinic for Hematology, University of Bochum, Minden, Germany; 14 Department of Hematology and Oncology, Red Cross Hospital Munich, Munich, Germany; 15 Medical Faculty, Department of Hematology, Oncology, Cell and Radiation Therapy, Otto von Guericke University Magdeburg, Magdeburg, Germany

**Keywords:** Central venous catheter, Removal, Central venous catheter-related bloodstream infection, Prevention, Male sex

## Abstract

**Purpose**

In patients with hematological malignancies and central venous catheters (CVC) men have a higher risk for CVC related bloodstream infections (CRBSI) than women. Hence, preventive strategies such as earlier CVC removal may reduce the overall CRBSI (oCRBSI) rate.

**Methods**

A randomized simulation using data from a CVC registry was performed. Data were derived from unselected patients with hematological or oncological diseases and a CVC. Patients were randomized to an experimental (EXP-G) or control group (CON-G). For the men in the EXP-G, one-day increments of an earlier CVC removal were simulated based on actual data and compared to men in the CON-G without simulation. The primary endpoint was oCRBSI rate.

**Results**

Underlying diseases were mainly hematological malignancies (87.3%). In the male patients with a median CVC in situ time of 13 days, the oCRBSI rate was 7.3% (103/1408), with 7.3% (53/729) in the EXP-G and 7.4% (50/679) in the CON-G. In the EXP-G, simulated shortening of CVC time by 1 day in men resulted in an oCRBSI rate of 5.2% compared with 7.4% in the CON-G (*p* = 0.12). Shortening CVC time by 2 days led to an oCRBSI rate of 4% (*p* = 0.008); the corresponding median CVC time was 11 days. A 3-day shortening further to 10 days reduced the oCRBSI rate to 2.7% (*p* < 0.001). Women from the same registry showed a 3.3% (30/920) oCRBSI rate after 12 days.

**Conclusion**

In conclusion, shortening the CVC dwell time in men by 2 or 3 days, corresponding to a CVC duration of 10–11 days, reduces the CRBSI rate in men and mitigates the higher risk, compared to women.

Clinical Trial Registration: SECRECY (Study to Evaluate Central Venous Catheter-related Infections in Hematology and Oncology) was registered within the German Clinical Trial Register (DRKS; no. DRKS00006551) on 29 September 2014.

**Supplementary Information:**

The online version contains supplementary material available at https://doi.org/10.1007/s15010-026-02888-7.

## Introduction

Bloodstream infections (BSI) are one of the most serious infectious complications in patients with cancer [1], occurring more frequently in those patients with hematological malignancies than in those with solid tumors [2]. Furthermore, men show a higher susceptibility to many infections, like BSI [3], with higher mortality rates than women [3]. An important source of BSI, especially in patients with cancer, are central venous catheters (CVC) [2]. A propensity score-matched study on 3024 patients with cancer, almost exclusively with hematological malignancies (96.4%) and internal jugular vein (JV) CVC (98.5%), found a higher rate (9.9% vs. 6.7%; *p* = 0.002) and incidence (7.0 vs. 4.7 per 1000 CVC days; *p* = 0.002), respectively, of CVC-related BSI (CRBSI) in men compared to women. In a multivariable regression, male sex proved to be an independent risk factor for CRBSI (hazard ratio 1.53 [95% confidence interval {95% CI} 1.19–1.96]; *p* = 0.001) [4]. The reason for this phenomenon is unknown so far and preventive measures in male patients should be studied. As CRBSI shows a time-dependent risk [4] interventions like earlier CVC removal in men may be the most effective intervention.

Conducting a prospective randomized controlled trial (RCT) in this specific cohort is challenging due to the clinical complexity associated with hematological malignancies and CVCs. Furthermore, the relatively low event rate would necessitate a large-scale, multi-center study with an extended recruitment period [5, 6]. Registry-based RCT (RRCT) are a good option. By using registries as a platform for recruitment, case records, data collection, randomization, and follow-up, RRCT are a cost-effective and popular methodology to answer comparative effectiveness clinical questions [7, 8]. To perform a hypothetical randomized study retrospectively, we conducted a target-trial-emulation (TTE) based on our registry data.

In order to investigate if a simulated intervention with only minimal shortening of CVC dwelling time in men leads to a lower overall CRBSI (oCRBSI) rate in the setting of patients with hematological and oncological diseases, we performed a TTE using data from a CVC registry. Time reduction was chosen to be feasible in clinical practice and to reach the oCRBSI rate of a comparable group of women taken from a registry.

## Methods

Data were derived from the German SECRECY registry (Study to Evaluate Central Venous Catheter-related Infections in Hematology and Oncology; described in detail elsewhere [9]) on CVC from non-selected patients from 13 participating hematology/oncology sites. Only patients with hematological or oncological diseases with JV CVC being ≥ 1 day in situ were included in the present analysis. CRBSI were assessed according to the 2012 Infectious Diseases Working Party (AGIHO) of the German Society of Hematology and Medical Oncology (DGHO) criteria [10] and summarized as the combination of *definite*/*probable* CRBSI (“CRBSI”). In brief, *definite* CRBSI required either a differential time-to-positivity ≥ 2 h in a pair of peripheral and central blood cultures or the detection of the same pathogen with the same susceptibility pattern in a peripheral blood culture and at the CVC tip. For *probable* CRBSI, one of the following scenarios needed to be fulfilled: (i) detection of the same pathogen typically causing CRBSI in a peripheral and central blood culture without fulfilling criteria of *definite* CRBSI and exclusion of other infection sites; (ii) exit site infection combined with BSI without criteria for *definite* CRBSI [10].

Neutropenia was defined as an absolute granulocyte count < 500/µL.

Consecutive patients were randomized using Sealed Envelope™ (a web-based platform for randomization and online databases for clinical trials (https://www.sealedenvelope.com); London, UK) and stratified per center, in a 1:1 ratio, to the experimental (EXP-G) and control group (CON-G). In the EXP-G, one-day steps of an earlier CVC removal were simulated in men based on actual registry data. Simulation was performed only for CVC indwelling for > 7 days. In the CON-G, no simulation was performed. The method of simulation of CVC removal (intervention; “What would have happened if...?”) was done in a manner of a modified and “reverse” landmark analysis that ignores all CVC days and CRBSI events after a defined landmark [8] (i.e., the actual day of CVC removal minus *x*-days; Supplementary Material, Fig. S1).

The primary endpoint was the oCRBSI rate, secondary endpoint was the oCRBSI incidence. Additionally, the number of CVC needed to be removed (NNR) to prevent one CRBSI in men [8] as well as the relative risk reduction (RRR) of CRBSI were calculated.

Sample size calculation (using Sealed Envelope™) was performed according to results from our previous study [4] aiming for a CRBSI rate reduction of 3.2% (from 9.9% in men to 6.7% in women). Based on a two-sided type I error α = 0.05, 2328 CVC were calculated to reach a power of 1-β = 0.8, meaning 1164 CVC per group (Table 1).

**Table 1:** Patients, CVC and CRBSI characteristics: descriptive comparison of men in experimental and control group

Parameter	Experimental Men, *n* = 729	Control Men, *n* = 679
Patients		
Median age (IQR), years	59 (49–66)	60 (51–67)
Underlying disease, *n* (%)		
Acute myeloid leukemia	198 (27.2)	203 (29.9)
Multiple myeloma	168 (23.0)	161 (23.7)
Non-Hodgkin lymphoma	157 (21.5)	133 (19.6)
Acute lymphoblastic leukemia	58 (8.0)	53 (7.8)
Hodgkin lymphoma	22 (3.0)	20 (2.9)
Myeloproliferative neoplasm	20 (2.7)	15 (2.2)
Myelodysplastic neoplasm	10 (1.4)	12 (1.8)
Germ-cell tumor	28 (3.8)	20 (2.9)
Sarcoma	20 (2.7)	14 (2.1)
Carcinoma	38 (5.2)	35 (5.2)
Benign hematological disease	10 (1.4)	13 (1.9)
Neutropenia at CVC insertion, *n* (%)	91 (12.5)	110 (16.2)
Neutropenia at CRBSI diagnosis, *n/N* (%)	49/53 (92.5)	45/50 (90.0)
Neutropenia at CVC removal, *n* (%)	191 (26.2)	196 (28.9)
CVC		
CHG-coated CVC dressing, *n* (%)	223 (30.6)	213 (31.4)
Antimicrobial-coated CVC, *n* (%)	149 (20.4)	127 (18.7)
Complicated CVC insertion, *n* (%)	48 (6.6)	65 (9.4)
CVC in situ (follow-up) time, days Median (IQR)	13 (5–20)	13 (6–20)
CRBSI		
CRBSI rate, *n* (%)	53 (7.3)	50 (7.4)
CRBSI incidence, *x*/1000 CVC days	5.2	5.2
Time to CRBSI onset, days Median (IQR)	13 (10–18)	13 (9–17)

*CHG* chlorhexidine gluconate, *CRBSI* central venous catheter-related bloodstream infection, *CVC* central venous catheter, *IQR* interquartile range

These two groups were split further in men and women with comparable characteristics to compare men with intervention to men without intervention (Supplementary Material, Table S1). The intention to compare men to women was to have a “benchmark” group and oCRBSI rate, as the aim of our intervention in men was to balance their increased oCRBSI rate to the lower risk of women and to compare men with and without intervention to exclude any sex-specific confounder.

Fisher’s exact test was used to evaluate differences in CRBSI rates, and incidence rate ratios were analyzed using the Mid-P exact test; continuous variables were compared by using the Mann-Whitney U test.

Statistical analysis was carried out using OpenEpi, version 3.01 (an open source software for epidemiologic statistics (https://www.openepi.com); Atlanta, GA, USA), MedCalc®, version 23.4.5 (a statistical software package for biomedical research (https://www.medcalc.org); Ostend, Belgium), and IBM® SPSS® Statistics, version 26 (Armonk, NY, USA). Two-sided *p*-values < 0.05 were considered statistically significant.

## Results

2328 CVC inserted between October 2020 and February 2025 were randomized (Supplementary Material, Fig. S1). Groups were separated into men and women, and men in the EXP-G (*n* = 729) and the CON-G (*n* = 679) were further analyzed in the first step. Underlying diseases were mainly hematological malignancies (Table 1). Neutropenia at the time of CVC insertion, removal and diagnosis of CRBSI showed no significant difference between the EXP-G and the CON-G. In the whole cohort, the oCRBSI rate was 7.3% (103/1408), with 7.3% (53/729) in the EXP-G and 7.4% (50/679) in the CON-G. Before simulation, groups were well balanced for all risk factors assessed by our registry. The median CVC dwelling time for men was 13 days in both groups (Table 1).

In the EXP-G, after simulation of shortening CVC dwelling time by 1 day in men, the oCRBSI rate was 5.2% compared to 7.4% in CON-G (*p* = 0.12) with a CRBSI rate for men of 5.2% compared to 7.3% before the intervention. The oCRBSI incidence was 3.9 vs. 5.2/1000 CVC days (*p* = 0.19).

Simulation of shortening CVC dwelling time by 2 days in men led to an oCRBSI rate of 4% (*p* = 0.008), an oCRBSI incidence of 3.1/1000 CVC days (*p* = 0.03). The corresponding median CVC dwelling time was 11 days (*p* < 0.001). For the simulation of a 3-day shortening in men, the oCRBSI rate was 2.7% (*p* < 0.001), the oCRBSI incidence 2.3/1000 CVC days (*p* = 0.001); median CVC dwelling time was 10 days (*p* = 0.001) (Table 2).

**Table 2:** Outcome analyses for simulated stepwise earlier CVC removal

Parameter Simulated CVC removal in men	Experimental Men, *n* = 729	Control Men, *n* = 679	*p*-value
1 day “earlier” CVC removal			
CVC in situ (follow-up) time, days			
Median (IQR)	12 (5–19)	13 (6–20)	0.04^a^
CRBSI rate, *n* (%)	38 (5.2)	50 (7.4)	0.12^b^
NNR, *n*	49		
RRR, %	28.3		
CRBSI incidence, x/1000 CVC days	3.9	5.2	0.19^c^
2 days “earlier” CVC removal			
CVC in situ (follow-up) time, days			
Median (IQR)	11 (5–18)	13 (6–20)	< 0.001^a^
CRBSI rate, *n* (%)	29 (4.0)	50 (7.4)	0.008^b^
NNR, *n*	31		
RRR, %	45.3		
CRBSI incidence, x/1000 CVC days	3.1	5.2	0.03^c^
3 days “earlier” CVC removal			
CVC in situ (follow-up) time, days			
Median (IQR)	10 (5–17)	13 (6–20)	< 0.001^a^
CRBSI rate, *n* (%)	20 (2.7)	50 (7.4)	< 0.001^b^
NNR, *n*	23		
RRR, %	62.3		
CRBSI incidence, *x*/1000 CVC days	2.3	5.2	0.001^c^

*CRBSI* central venous catheter-related bloodstream infection, *CVC* central venous catheter, *IQR* interquartile range, *NNR* number of CVC needed to be removed to prevent one CRBSI, *RRR* relative risk reduction

^a^Mann-Whitney U test

^b^Fisher exact test

^c^Mid-P exact test

The corresponding NNR values were 49, 31, and 23, respectively, and the corresponding RRR values were 28.3%, 45.3%, and 62.3%, respectively (Table 2).

The comparable group of women from our registry (Supplementary Material, Table S1) with balanced risk factors to the EXP-G and CON-G showed an oCRBSI rate of 3.3% (30/920) and oCRBSI incidence of 2.3/1000 CVC days after 12 days.

Simulation was additionally done for both groups, taking men and women together to describe the risk reduction for the whole group and estimate the CVC removal timepoint, when the risk of the man reaches the risk of a comparable group of women.

In the whole cohort, the oCRBSI rate was 5.7% (133/2328), with 7.3% (103/1408) in men and 3.3% (30/920) in women. The median CVC dwelling time for men in the EXP-G was 13 days compared to 12 days for women (*p* = 0.92) in the CON-G.

In the EXP-G, after simulation of shortening CVC dwelling time by 1 day in men, the oCRBSI rate was 4.5% compared to 5.7% in CON-G (*p* = 0.21). The oCRBSI incidence was 3.2 vs. 4.0/1000 CVC days (*p* = 0.28). Simulation of shortening CVC dwelling time by 2 days in men led to an oCRBSI rate of 3.7% (*p* = 0.03) and an oCRBSI incidence of 2.8/1000 CVC days (*p* = 0.06). The corresponding median CVC dwelling time was 11 days (*p* = 0.03). For the simulation of a 3-day shortening in men, the oCRBSI rate was 2.9% (*p* = 0.001), the oCRBSI incidence was 2.3/1000 CVC days (*p* = 0.007), median CVC dwelling time was 10 days (*p* = 0.001) (Supplementary Material, Table S2).

## Discussion

CVC-related infections in patients with cancer are common and are associated with high morbidity and potentially severe sequelae (e.g., endocarditis, septic thrombosis, abscess formation, osteomyelitis [11]) which can disrupt anti-malignancy treatment plans. Men carry a substantially increased risk, with an approximately 1.5-fold higher CRBSI rate compared with women, reported in a previous study [4] and more than a 2-fold risk of CRBSI in the current study. Against this background, targeted preventive measures – especially those addressing sex-specific risk factors – are urgently needed to reduce CRBSI incidence.

As the reasons for sex-specific differences still remain unclear; we focused on well-known and modifiable risk factors such as CVC dwelling time and evaluated whether shortening dwell time in men for a short period could reduce CRBSI incidence.

Intensive care unit patients with CRBSI showed a significantly longer mean CVC time of 22 days compared to 12 days in patients without CRBSI (odds ratio 1.95 [95% CI 1.21–2.13]; *p* < 0.001) [12]. A *post-hoc* analysis showed an increased risk of CRBSI with an exponential increase after more than 10 days after CVC insertion [13]. No longer needed, the CVC should thus be removed in order to reduce the risk of CRBSI [11].

A predefined, time-based removal of CVC to prevent CRBSI might reduce this risk. However, this approach is not always feasible in clinical practice. Many patients – especially those with hematological malignancies – require ongoing venous access for delivering chemotherapy, blood products, anti-infective agents or parenteral nutrition and the clinical course can be unpredictable. Consequently, clinicians may be unable to remove a “needed” CVC at a fixed time point, limiting the applicability of routine scheduled removals and necessitating pragmatic, risk-adapted strategies. Moreover, a previous registry-based simulation found that scheduled CVC removal was effective for CRBSI prevention only after 7 days after insertion but not after 14 days [8], further underscoring the limited benefit of fixed-time removals in this population.

Assessment of the ongoing necessity for a CVC is mandatory and daily documentation of critical evaluation for removal should be documented [14]. Data on the adherence to existing guidelines are missing in this patient cohort. Nevertheless, from our experience and extrapolated from intensive care unit data, reduction of CVC dwelling time is possible by multimodal intervention [15, 16].

However, based on data from the present analysis, it appears justified to remove CVC 2–3 days earlier in men than originally planned/pre-defined. This approach could easily be implemented in clinical practice for selected patients not strictly requiring a CVC (Supplementary Material, Fig. S3). Special attention should be focused on CVC in men after 10 days of dwelling time, as there is more than a doubling of the CRBSI rate in the next 3 days.

Of note, shortening the CVC time by 2–3 days not only resulted in a significantly lower CRBSI rate, but also in a lower CRBSI incidence in men, comparable to women without intervention. The exact time of earlier CVC removal in men is between days 2 and 3, as the risk for men falls under the risk of the whole group between these time points.

Simulation was first done with groups, consisting of men and women with only intervention for men in the EXP-G to test the effect on the whole group and estimate the timepoint, when the risk for men and women becomes equal with a time-based intervention in men. This approach can not differentiate between time- and sex-specific effects between both groups. Therefore, simulation was done additionally with groups of only men to exclude any potential sex-specific confounder as far as possible.

The RRR values for men were high, the corresponding NNR as well (*n* = 23 for the shortened CVC time of 3 days (Table 2)). The latter is not unexpected, the overall CRBSI rate in our study was low at 5.7%, which explains the high NNR values. Double digit numbers needed to treat – or NNR – values are usually considered unimportant with respect to routine efficacy measures but may become important regarding adverse outcomes with long-term consequences [17]. In patients with hematological malignancies, CRBSI are associated with high morbidity [18], potentially resulting in complications such as endocarditis, septic thrombosis, abscess formations, osteomyelitis, or relapse of infection [11]. The NNR values determined in this study should therefore be considered relevant.

The study analyzed CRBSI in men and women. It should be noted that the parameter “sex” was determined by the data given in patients’ health insurance records, representing the “legal gender”. Maybe there is an overlap with the biologically informed parameter “sex,” but it was not possible to disentangle it fully from the self-assigned parameter “gender” [4].

Limitations for the current analysis include the retrospective character and CVC-focused registry data. We therefore cannot fully exclude other time-dependent confounders and imbalances between the CON- and EXP-G.

In conclusion, in patients with hematological and oncological diseases and JV CVC, shortening the CVC time in men by 2–3 days, corresponding to a CVC time maximum of 10–11 days, reduces the CRBSI rate in men to the level of women and results in a significantly lower oCRBSI rate. Greater effort should be directed toward identifying sex-specific risk factors for CRBSI.

## Supplementary Information

Below is the link to the electronic supplementary material.

Supplementary Material 1

## Data Availability

The datasets used and/or analyzed during the current study are available from the corresponding author on reasonable request.

## Declarations

### Generative AI in scientific writing

No generative artificial intelligence (AI) and AI-assisted technologies were used in the writing process.
