# Supplementary material for: Shortening of central venous catheter time in men with hematological and oncological diseases for prevention of central venous catheter-related bloodstream infections: a target-trial-emulation study
Source: Infection. 2026 Jul 10;54(5):2969–75. doi: 10.1007/s15010-026-02888-7 (PMC13727808; doi:10.1007/s15010-026-02888-7)
Supplement: Supplementary file 1 [file 15010_2026_2888_MOESM1_ESM.docx]

**Supplementary Material**

**Figure S1.** Example of the procedures and respective simulation analysis carried out for a 1-day “earlier” CVC only in men in the experimental group. Dots represent time points of CVC removal in reality. Crosses with rings represent time points of CRBSI diagnosis. By shortening CVC time by 1 day, all CVC days and CRBSI events are ignored after this landmark.

**Abbreviations:** CRBSI, central venous catheter-related bloodstream infection; CVC, central venous catheter.

**Figure S2.** Registry and study enrollment, respectively, and randomization.

^a^Non-selected, chronologically according to CVC insertion date.

^b^Multiple reasons are possible.

^c^The registry consisted of only six sites during 2013-2020.

^d^Differences in both groups due to randomization/stratification per center.

**Abbreviation** CVC, central venous catheter.

**Figure S3.** Example of implementing study results into clinical practice. A man with multiple myeloma and high-dose chemotherapy with autologous hematopoietic cell transplantation, with initial febrile neutropenia, later defervescence and white blood cell regeneration on Thursday and planned discharge on Monday. Shortening the CVC time by 3 days (CVC removal before the weekend; with relative risk reduction for CRBSI of more than 60%), corresponding to a CVC time of 11 days.

**Abbreviations** ANC, absolute neutrophil count; CRBSI, central venous catheter-related bloodstream infection; CVC, central venous catheter.

**Table S1.** Patients, CVC and CRBSI characteristics: descriptive comparison of men and women

| **Parameter** | **Experimental**  ***n*=1166** | | **Control *n*=1162** | |
| --- | --- | --- | --- | --- |
|  | **Men**  ***n*=729** | **Women**  ***n*=437** | **Men**  ***n*=679** | **Women**  ***n*=483** |
| **Patients** |  |  |  |  |
| Median age (IQR), years | 59 (49-66) | 61 (50-67) | 60 (51-67) | 60 (48-67) |
| Underlying disease, *n* (%)  Acute myeloid leukemia  Multiple myeloma  Non-Hodgkin lymphoma  Acute lymphoblastic leukemia  Hodgkin lymphoma  Myeloproliferative neoplasm  Myelodysplastic neoplasm  Germ-cell tumor  Sarcoma  Carcinoma  Benign hematological disease | 198 (27.2)  168 (23.0)  157 (21.5)  58 (8.0)  22 (3.0)  20 (2.7)  10 (1.4)  28 (3.8)  20 (2.7)  38 (5.2)  10 (1.4) | 156 (35.7)  113 (25.9)  78 (17.8)  31 (7.1)  10 (2.3)  9 (2.1)  5 (1.1)  0  5 (1.1)  16 (3.7)  14 (3.2) | 203 (29.9)  161 (23.7)  133 (19.6)  53 (7.8)  20 (2.9)  15 (2.2)  12 (1.8)  20 (2.9)  14 (2.1)  35 (5.2)  13 (1.9) | 174 (36.0)  109 (22.6)  89 (18.4)  34 (7.0)  11 (2.3)  6 (1.2)  8 (1.7)  0  7 (1.4)  29 (6.0)  16 (3.3) |
| Neutropenia at CVC insertion, *n* (%) | 91 (12.5) | 74 (16.9) | 110 (16.2) | 72 (14.9) |
| Neutropenia at CRBSI diagnosis, *n/N* (%) | 49/53 (92.5) | 13/14 (92.9) | 45/50 (90.0) | 14/16 (87.5) |
| Neutropenia at CVC removal, *n* (%) | 191 (26.2) | 120 (27.5) | 196 (28.9) | 135 (28.0) |
|  |  |  |  |  |
| **CVC** |  |  |  |  |
| CHG-coated CVC dressing, *n* (%) | 223 (30.6) | 122 (27.9) | 213 (31.4) | 139 (28.8) |
| Antimicrobial-coated CVC, *n* (%) | 149 (20.4) | 75 (17.2) | 127 (18.7) | 107 (22.1) |
| Complicated CVC insertion, *n* (%) | 48 (6.6) | 45 (10.3) | 65 (9.4) | 52 (10.8) |
| CVC *in situ* (follow-up) time, days  Median (IQR) | 13 (5-20) | 12 (5-20) | 13 (6-20) | 12 (5-20) |
|  |  |  |  |  |
| **CRBSI** |  |  |  |  |
| CRBSI rate, *n* (%) | 53 (7.3) | 14 (3.2) | 50 (7.4) | 16 (3.3) |
| CRBSI incidence, *x*/1000 CVC days | 5.2 | 2.3 | 5.2 | 2.4 |
| Time to CRBSI onset, days  Median (IQR) | 13 (10-18) | 12 (10-20.25) | 13 (9-18.75) | 12 (5.25-18.5) |

**Abbreviations:** CHG, chlorhexidine gluconate; CRBSI, central venous catheter-related bloodstream infection; CVC, central venous catheter; IQR, interquartile range.

**Table S2.** Outcome analyses of cumulated groups with intervention of men in the EXP-G

| **Parameter**  **Simulated CVC removal in men** | **Experimental**  ***n*=1166** | **Control *n*=1162** | ***p-*value** |
| --- | --- | --- | --- |
| **1 day “earlier” CVC removal**  CVC *in situ* (follow-up) time, days  Median (IQR)  CRBSI rate, *n* (%)  CRBSI rate for men, *n*/*N* (%)  NNR for men, *n*  RRR for men, %  CRBSI incidence, *x*/1000 CVC days | 12 (5-19)*  52 (4.5)  38/729 (5.2)  49  28.3  3.2 | 12 (5-20)*  66 (5.7)  4.0 | 0.25^a^  0.21^b^  0.28^c^ |
| **2 days “earlier” CVC removal**  CVC *in situ* (follow-up) time, days  Median (IQR)  CRBSI rate, *n* (%)  CRBSI rate for men, *n*/*N* (%)  NNR for men, *n*  RRR for men, %  CRBSI incidence, *x*/1000 CVC days | 11 (5-18)*  43 (3.7)  29/729 (4.0)  31  45.3  2.8 | 12 (5-20)*  66 (5.7)  4.0 | 0.03^a^  0.03^b^  0.06^c^ |
| **3 days “earlier” CVC removal**  CVC *in situ* (follow-up) time, days  Median (IQR)  CRBSI rate, *n* (%)  CRBSI rate for men, *n*/*N* (%)  NNR for men, *n*  RRR for men, %  CRBSI incidence, *x*/1000 CVC days | 10 (5-17)*  34 (2.9)  20/729 (2.7)  23  62.3  2.3 | 12 (5-20)*  66 (5.7)  4.0 | 0.001^a^  0.001^b^  0.007^c^ |

**Abbreviations** CRBSI, central venous catheter-related bloodstream infection; CVC, central venous catheter; IQR, interquartile range; NNR, number of CVC needed to be removed to prevent one CRBSI; RRR, relative risk reduction.

*Stated and compared men in the experimental group and women in the control group.

^a^Mann-Whitney U test.

^b^Fisher exact test.

^c^Mid-P exact test.
